# Understanding the Impact of Generation Z on Risk Management—A Preliminary Views on Values, Competencies, and Ethics of the Generation Z in Public Administration

**DOI:** 10.3390/ijerph19073868

**Published:** 2022-03-24

**Authors:** Zbysław Dobrowolski, Grzegorz Drozdowski, Mirela Panait

**Affiliations:** 1 Institute of Public Affairs, Jagiellonian University, 30-348 Kraków, Poland; 2 Department of Economics and Finance, Jan Kochanowski University, 25-369 Kielce, Poland; grzegorz.drozdowski@ujk.edu.pl; 3 Department of Cybernetics, Economic Informatics, Finance and Accounting, Petroleum-Gas University of Ploiesti, 100680 Ploieşti, Romania; mirela.matei@upg-ploiesti.ro

**Keywords:** generation Z, competencies, risk, public administration, ethic

## Abstract

Gen Z, people born in the Internet age, are entering the labour market and soon will be responsible for public administration. Such a situation creates the need to study their professional motivations and competencies. We aim to determine: (1) What are the motivating factors of Gen Z representatives? (2) What is the factor’s structure of competencies of Gen Z employees? (3) Do Gen Z’s interest in public administration result from their needs to realise the public interest? These questions are fundamental for the strategy of hiring and training newcomers. This original paper’s insights have emerged iteratively based on a systematic literature searching method and data obtained from the surveys (*n* = 335). Research of Polish representatives of Gen Z showed that their expectations are similar to those found in other countries. However, their responses suggest that ethical issues are not the most important for them. The presence of generation G on the labor market will generate a paradigm shift in the activity of companies and public institutions that will be the employers of these young people. Reconfiguration of the principles of human resource management is necessary so that organizations benefit from the qualities of generation Z—they gravitate towards gamified processes because of mobile-centricity; they are natives of global communication, self-learners, and self-motivators; they appreciate transparency.

## 1. Introduction

One may generalise that the issue of matching motivational tools to different generations is well recognised, and there are many studies on generational differences. However, the continuation of the study on Generation Z (Gen Z) can bring other several contributions. First, like other researchers, e.g., [1,2], we think that such a study may enrich theorists’ and practitioners’ awareness of generational differences. Moreover, better understanding the Gen Z expectations and their features allows decision-makers to fit workplaces better and manage different risk types, including human resource management. Such knowledge helps avoid a job mismatch and reduce the risk of resources waste [3]. Finally, it may help maintain skilful young employees [4] and develop workplaces desired by Gen Z.

During this study, we took into account the Mannheim (1952) theory of generations [5] and References [1,6,7]. We assume that new generations are usually described as being around 17–20 years in length because this time enables mapping cultural changes [8,9]. We also assume that Gen Z comprises people born after 1995 [10] or, according to some researchers, after 2000 [11].

Most studies on Generation Z have been carried out from the perspective of these young people as consumers and their attractiveness for consumer goods companies and retailers. In addition, given their digital capabilities, Generation Z has a global vision of economic and social phenomena, which is why they are increasingly concerned with promoting new concepts such as corporate social responsibility and sustainable development. Aware of the contribution they can make to sustainable development, Generation Z is increasingly involved as a volunteer in CSR companies promoting various companies that seek to either reduce the negative externalities generated by economic activity or to improve social and environmental performance [12,13]. Generation Z awareness of the important role that companies can play in promoting the principles of sustainable development generates an improvement in their behaviour as consumers, which will avoid wasting food and energy, reduce the purchase of less necessary goods, and sanction the actions of companies with a negative impact on the environment. According to Seabra et al., 2021 [14] “Gen Z is environmentally aware and concerned, and value an eco-friendly and healthy lifestyle” (p. 11). Considering the education received in school, the representatives of generation Z have at their disposal complex skills and knowledge related to entrepreneurship, financial market, food, sustainable development, etc. Therefore, they “have both the resources and willingness to influence the destiny and operation of CSR” [15] (p. 3). Understanding the behaviour of Generation Z to promote sustainable development is essential not only for companies but also for public authorities who can thus shape social and economic policies and adopt regulations to encourage the involvement of young people in CSR actions carried out by companies on a voluntary or mandatory basis. Given that Generation Z is geared towards digital media, these young people spend more time looking for the information posted on public portals or social media, and the responsible behaviour of companies is very closely monitored and appreciated [16,17]. These consumers are very vigilant and very well informed, which is why they quickly sanction any detected greenwashing attempt [18,19,20,21,22]. The main studies published in the international stream are focused on Gen Z behaviour as consumers. Very few studies focus on Generation Z as employees [12,23], one of the reasons being that these young people are in the process of completing their studies and starting their professional careers, and few of them are already present on the labour market. For this reason, this study is individualized considering that the research follows the behaviour of Gen Z as employees who will work in the central and local public administration, whose task will be not only (i) to develop economic and social policies in the context of the transition to low carbon economy but also (ii) to set up specific tools for the application of these policies. Moreover, these policies must be thought of and implemented in an increasingly complex context given the phenomenon of globalization, the intensification of black swan events, the increased risk of cyberattacks, and the need to manage climate change.

Generation Z is considered to be much more motivated and determined to achieve its goals. According to [24] generation Z is “more oriented to entrepreneurship, have grown up with the search engines and they like to discover content that meets their needs”. These qualities will certainly contribute to a better implementation of the proposed measures at the level of central and local public authorities, considering the quality of these young as civil servants. However, studies [12,23] have also shown the existence of weaknesses such as their desire to earn promotion quickly, the desire to occupy important positions, and the inclination to perform individual tasks and more activities that require teamwork.

We aim to determine: (1) What are the motivating factors of Gen Z representatives? (2) What is the factor’s structure of competencies of Gen Z employees? (3) Do Gen Z’s interest in public administration result from their needs to realise the public interest? These questions are fundamental for the strategy of hiring and training newcomers and mitigating risk in public administration. This paper’s insights have emerged iteratively based on the systematic literature searching method and data obtained from the surveys (*n* = 335). The survey was run in Poland among students. The focus of the study on student behaviour was based on several considerations. The study aims to identify the implications of hiring young people in the Z generation in public administration, which is why students were selected as a population for this research with high chance that they have employment after graduation. In addition, given the metamorphosis of the mission of universities, higher education institutions are increasingly involved in the development of their communities by providing increasingly complex entrepreneurial skills to students as well as initiating and supporting student start-ups. Therefore, compared to other young people in generation Z, students have superior entrepreneurial skills acquired during their studies, regardless of their specialization. Promoting the principles of sustainable development is achieved at the university level on several levels, both by introducing specific disciplines in the curriculum, such as Sustainable Development, Corporate Social Responsibility, and Business Ethics, and by pursuing a more responsible behaviour from higher education institutions. to all stakeholders (environment, local communities, public authorities). Therefore, students acquire skills in environmental protection or involvement in local communities during their studies. The tendency towards massification in higher education [25,26,27] means that a large part of the Z generation are students, which is why the literature identified has focused on students as a representative sample of the Z type [14,28,29]. This study is distinguished by the novelty of the approach, namely, Gen Z involvement in public administration; this fact being favoured by the responsible behaviour of these young people and their inclination to protect the environment and involvement in local communities. Given these considerations coupled with their ability to react quickly and rapid access to information, young people from Generation Z will be specialists who will promote the implementation of SDGs not only in public institutions and also contribute to the proper design of public policies to enhance responsible social behaviour of companies in various fields. The increase in uncertainty and the occurrence of black-swan-type events generate more and more risks in various fields, which must be managed primarily by public authorities who must find specific measures and tools to help companies and other stakeholders to overcome situations. For these reasons, the involvement of Generation Z in public administration is becoming a topic of interest not only for researchers but also for educational institutions that need to provide them with the specific skills and knowledge to cope with an increasingly volatile, complex, and dynamic environment.

For the best possible presentation of our research results, we proposed the following structure for the article. First, we review theories related to Gen Z. Second, we show research findings. Finally, conclusions and potential research opportunities are given.

## 2. Literature Review

Knapp, Weber, and Moellenkamp (2017) aptly noted that people representing Gen Z would soon be new employees on a massive scale. Is this fact, coming from natural generation change, is essential for public administration? The answer to the above question is positive [30]. The authors [31,32,33,34,35,36,37,38,39,40,41,42] emphasise the uniqueness of Gen Z in their daily use of social media and the need to share their experiences, expectations, and views with others. Some authors pointed out that young employees are labelling their expectations as excessive and may not be well prepared for workplace realities [43,44]. Some researchers argued that the members of Gen Z tend to have higher awareness and concern about environmental issues, and therefore, they may better react to green policies [44,45,46]. Another group of researchers did not confirm this assumption [47,48,49]. Different research conclusions about Gen Z show that it is too early to generalise Gen Z features [50,51]. Other researchers also underlined that it is easy to stereotype younger employees [52,53] and that it is too early for certain generalisations. We follow the view of Urick et al. (2017) that a lack of comprehensive research of Gen Z may lead to practitioners’ lower understanding of Gen Z needs and to accept that everything that has already been said about generation Z seems premature [2]. Based on the literature analysis, it is possible to generalise that although Gen Z is presented in the literature from various perspectives, this generation and its impact on public administration are still not fully recognised.

Research on Gen Z is crucial because this generation is entering the job market. For example, in the US, this generation will make up about 20 per cent of all employees. In other countries, depending on the birth rate, the share of Gen Z in the labour market may be lower [37]. Employees of the young generation adopt the organisational culture, but also, due to the differences between them and older employees, bring new values to workplaces. They will also be responsible for risk management, which for this research was defined as a set of activities aimed at preventing the failure to achieve organisational goals. In formulating this definition, we know the complexity of risk management and a significant amount of research on this area of organisational activity [54,55,56,57,58,59,60,61,62,63,64,65].

**Figure 1:**
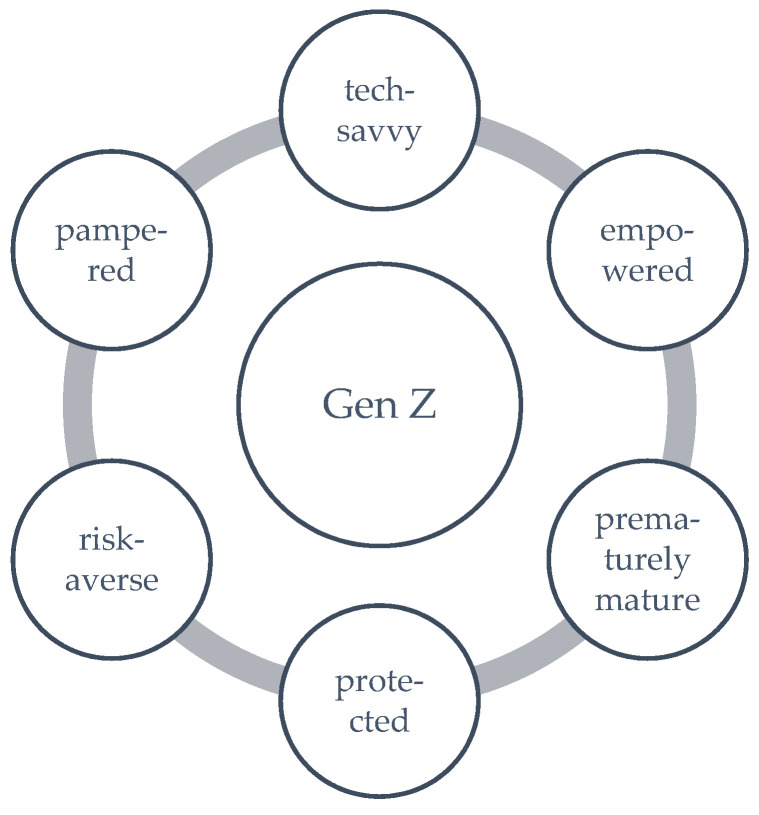
Gen Z characteristics.

Representatives of Gen Z, born after 1995, also known as “Digital Natives”, being in families with Gen X, started their life after communism fell in Central and Eastern Europe, some Asian and African countries and into a world facing global terrorism and globalisation. They are familiar with widespread electronic devices and digital technologies linked with e-social networking sites. In addition, “Generation Z has grown up with the mindset that risk is unacceptable. Members of Gen Z are more cautious and risk-averse than their parents” [66] (p. 81). Gen Z, characterised as tech-savvy, globally connected (in the virtual world), and agile, is recognised based on the environment in which it grows and some characteristics [32], sometimes with a pejorative sound, such as in Figure 1.

Some other names of Gen Z that one may find are the following: iGeneration, Gen Tech, Gen Wii, Net Gen, Digital Natives, Plurals, and Zoomers [34]. The new generation was born in the era of the Internet and mobiles. People from Gen Z have access to resources that Gen Y did not at that age, including websites to teach themselves new skills. They have access to e-programs that Gen Y did not [32]. There is a need to determine whether people representing Gen Z constitute a different category of employees? Considerations in this area should begin by pointing out that this is only one generation born in the environment of the Internet, e-games, e-media, and globalisation. It is pointed out by, among others [67]. They state that games influence this generation by forming neural pathways. Games reinforce certain beliefs about the self, how the world should work, how people relate to one another, and finally, life’s general goals. Games create a self-centred universe where players can influence other people and objects [67,68].

Research conducted in India showed that people from Gen Z would like to work for firms that do not bother about working hours, leaves, and permissions. They felt responsible and did not want somebody else to tell them what to do. They waited for feedback as a tool for improvement but when needed. They would treat a friend at work rather than a boss, which positions them into agile companies. They underlined freelancing or doing something on their own. People from Gen Z saw a career as an opportunity to develop their own life and perceived materialism through the prism of symbols of a “good life”. They wanted to imbibe global values and be seen as somebody who could influence globally. Many Gen Z people did not see employment permanence as a value. They believed that it was worth checking employment opportunities in various companies. People representing Gen Z use e-media often. They visit YouTube multiple times per day, Twitter, Google, and Instagram. They like sharing their knowledge and opinions with others [32].

Usage of e-media makes faster information change and creates a basis for agile organisations, which is particularly important in security issues [58]. In addition, compared to the previous generation Y, people from Gen Z have an even greater ability to multitask. They are creative, innovative, and optimistic. People representing Gen Z prefer independent work, often stay in virtual space, and prefer communication using abbreviations [37,69]. Gen Z consists of active problem-solvers, independent learners, and people following social justice and sustainability [34,70]. Because Gen Z is accustomed to receiving information on demand and very quickly, they may procrastinate until the last five minutes to complete tasks and expect managers to be available 24/7 for questions. Although they are adept at finding information, they may not analyse it for valid evidence. They lack skills to evaluate information critically and require this training via engaging ways (e.g., journaling, discussion, and reflection and learning through teamwork, debate, problem-solving, and reflection) [70,71,72]. Risk management is widely presented in the literature [54,56,73,74,75,76,77,78]. Researchers point out that risk management starts with analysing all crucial information. Together with the risk appraisal, this information forms the input material upon which risk management options are assessed, evaluated, and selected. Risk management can be perceived as a part of management control in public administration. Such a situation exists in Poland, where the public administration must manage risk according to public finance law. It includes but is not limited to ethical issues (creating, maintaining, and promoting ethics in public administration).

Considering the results of the main studies presented in this section and focused on Gen Z behaviour, there are the following research questions: (1) What are the motivating factors of Gen Z representatives? (2) What is the factor’s structure of competencies of Gen Z employees? (3) Do Gen Z’s interest in public administration result from their needs to realise the public interest? In addition to surveys, we used the systematic literature searching method (Hart, 2001) to have an extensive theoretical foundation. Using interpretations based on previous research leads to new insights on essential research issues [79].

## 3. Methods

We assumed that our research focused on Gen Z competencies belongs to social sciences. Therefore, this study does not always have to be reflected in the formalised language of mathematical logic and does not lead to the construction of unchanging theories but remains socially and historically limited to generalisations [80]. (However, the created concepts must be based on commonly shared cognitive assumptions referred to as the paradigm) [81]. We used the Burrell and Morgan (2017) classification of paradigms (widely recognised by researchers) to consider which research strategy fits our study [82]. We chose the strategy of epistemological pluralism, having an opportunity to use approaches drawn from different paradigms to obtain cognitive results. Therefore, we use functionalist and interpretative concepts (meaning and interpretation). We chose this methodology based on methodological triangulation to obtain a broader context of the studied issues [83,84].

Nordqvist and Gardner (2020) and Short and Payne (2020) recently discussed how literature could inspire the research [85,86]. We took their approach. We used the systematic review of the literature included several phases, starting from determining the purpose of the research, selecting the primary literature, selecting publications, using keywords, developing a database, and applying bibliometric and content analysis. We analysed 147 publications using the research databases and then selected (taking into account research problems) the publications listed in this article’s bibliography. We think that analysis of previous research leads to insights on essential research issues [79,87,88,89]. We also used the surveys to obtain the information necessary to resolve identified research problems. The survey lasted six weeks, and the respondents answered the questionnaire online from November 2021. We used Google Forms (Jagiellonian University, Kraków, Poland) and the Teams application. The study was anonymous in nature. The study included people representing Gen Z living in Poland—students. Three hundred thirty-five individuals expressed a willingness to answer the survey questions, and 34.5 per cent of all surveyed live in a rural area, the rest in cities.

## 4. Findings

The respondents were asked to answer several closed questions about their motivating factors in the workplace. The survey’s first question concerned the assessment of the values that the representatives of Gen Z consider to be the most important in their lives. The respondents’ answers are presented below in Table 1.

The next question concerned the social media used by representatives of Gen Z. The answers that the respondents could give were as follows (the respondents could give multiple answers)—Table 2 and Table 3. Nobody has stated that they are not using social media.

Our findings on the value of Gen Z and the use of social media in their lives align with the results of other researchers [31,32,33,34,35,36,37,38,39,40,41,42]. However, our research has shown a complete lack of values such as patriotism among the values of Generation Z.

In the next question, the respondents were asked to indicate factors that motivate them to work. Some respondents said that their wages (44.6 per cent) motivated them to work. The next factors were the following: friendly atmosphere in the workplace (44.9 per cent) and friendly relations with the immediate supervisor (8.4 per cent). The respondents expect from their workplace: fair and equal treatment (59.6 per cent), fast professional development (27.4 per cent), openness to the needs of others (10.5 per cent), and other expectations (5.1 per cent). Therefore, the next research results related to ethical issues are interesting (Table 4)

Others did not know how to answer the question about the role of ethics in the workplace. The survey’s answers concerning the reasons why they expressed interest in future work in public administration (Table 5) are shown below.

The next question concerned the structure of competencies of Gen Z representatives. The structure of competencies is presented in Table 6.

Finally, 45.3 per cent of respondents said that they prefer privacy, so they would like to have their own room in the workplace without sharing it with others; 12.7 per cent of respondents said that they want to work in the open space formula, and 42.0 per cent of the respondents stated that they do not care whether they will share their workspace with others or have their rooms in the workplace.

## 5. Discussion

Interpreting the research results, one may conclude the leading features from the competency profiles of the studied Gen Z representatives. From ten such features identified in the literature, we found that the competency structure of the analysed sample size of Generation Z representatives is based on five crucial features: commitment, creativity, flexibility, emotional balance, and activity. The lowest competency component included risk-taking, knowledge and skills, ethics, and leadership skills. The study was conducted on young people under 24 years of age and thus generally professionally inexperienced. The surveyed people are students, and therefore, their lower estimation of knowledge and skills seems to be proper at this stage of their personal development. This assessment and the next ones about risk-taking and leadership skills testify to the answers’ realism, self-criticism, and reliability. Research of Polish Gen Z representatives has shown that their expectations are similar to those found in other countries. Gen Z employees expect fair and partner treatment from their employers. They expect fair wages. For Generation Z, it is important to feel part of one big community. Their answers suggest a balanced approach to development. In addition, our findings confirmed previous research on critical components of competencies.

We discovered that the highest-rated traits of our surveyed Gen Z representatives were commitment, creativity, flexibility, and activity. It can be assumed that paying attention to this set of traits is indirectly connected with the need for self-realisation and the need for recognition and belonging. These findings confirm previous studies on this topic [32,34,37,69,70].

The qualities presented above refer, on the one hand, to the area of self-realisation and, on the other hand, to the ability to go beyond the usual schemes. Generation Z tries to create their solutions differently. It can be assumed that creating new solutions is an essential element of work in business. However, public administration has to follow administrative procedures and administrative law. Therefore, there is a need to take this issue during the training of newcomers, representatives of Gen Z.

We found a surprising dichotomy in the assessment of the ethical factors. Respondents rated their ethical competencies relatively high. They also expect from their workplace fair and equal treatment. However, surprisingly, they do not perceive ethical values as the most important in their workplaces. The virtualisation of social life makes it easier to make connections, but at the same time, it promotes the avoidance of responsibility for relationships. Our results confirmed previous research on cultural determinants of motivating factors [90,91,92]. Our study focused on the interest of surveyed Gen Z representatives in work in public administration showed that they result from the stability of employment and other public sector benefits. They also pointed out a lack of business skills. Only about one fifth surveyed pointed out the goal of public administration—the realisation of the public task as a crucial factor convincing them to work in public administration. In addition, some of them indicated that high salary is an essential factor in their lives. Meanwhile, wages in the public sector are not high and even lower than in the private sector in many posts.

Expectations of high wages in administration where this sector is generally not an attractive employer proves the lack of knowledge about the real world outside the university. Our study confirmed that young employees are labelling their expectations as excessive and may not be well prepared for workplace realities [43,44]. In addition, relatively little interest in public service and the perception of ethics as a secondary value can catalyse risk. The answers of Gen Z representatives may indicate the avenue of future training for newcomers. Ethical competencies and public service have to be enhanced because they are necessary to create social capital, fundamental for the stable realisation of public tasks and business operations [93,94,95,96,97,98,99]. The inclination of young people from generation Z towards promoting the principles of sustainable development as consumers will be reflected in their behaviour as employees, the skills acquired in volunteering being essential for future jobs. These young people will be able to design specific measures and economic policies in sustainable development, energy transition, or corporate social responsibility taking into account both the practical experience gained through the volunteer actions they participated in and the knowledge gained through various information channels. It will also increase the adaptability and flexibility of public institutions to the challenges posed by economic and social uncertainty. Generation Z youth will therefore be very valuable assets in public institutions and will increase the capacity of these entities to cope with uncertainty and black swan events [100,101,102,103,104,105,106].

## 6. Conclusions

The presence of generation Z on the labour market will generate a paradigm shift in the organization of activity at the level of companies and public institutions that will be the employers of these young people. Reconfiguration of the principles of human resource management is necessary so that organizations benefit from the qualities of generation Z—they gravitate towards gamified processes because of mobile-centricity; they are native of global communication, self-learners, and self-motivators, and they appreciate transparency and honesty.

The main limitation of the research is generated by the choice of Poland as the basis for the selection of Generation Z representatives who were the subject of the study. This was generated by the authors’ desire to obtain and use primary data, the study allowing the identification of specificities for generation Z in this country.

The present study helps to open avenues for further research. We believe that there is a need to determine whether Gen Z is ready to operate in uncertainty. We did not analyse this topic during our study. Meanwhile, the COVID-19 pandemic showed that public administration might operate in an uncertain environment more often. In addition to the identified limitations of our study, one may also add that more respondents should participate in future research to have more precise final research results. We also think there is a need to continue further study on patriotism and Gen Z, primarily because little is known about this feature of Gen Z in different countries and its influence on public administration. Finally, there is a need to recognize the structure of competencies of Gen Z in other countries.

Although this study focused on Gen Z in Poland, it may inspire research in other countries or members of the European Union. Moreover, studies on different fields of activity can be carried out to identify the specifics of the generation Z workforce in sectors such as industry or services. Our findings should be particularly interesting for decision-makers, who may use motivator’s tools dedicated to Gen Z employees, who will soon decide about successes or failures in daily operations. Public managers should also consider risk Gen Z employees’ behaviour in risk management. For example, in the self-assessment of management control systems, where ethical issues play an essential role.

The scientific conclusions presented in the paper aim to balance the processes that shape the competencies of Generation Z.

1. The need for a structured approach to research the factor structure of Generation Z competencies in public administration was justified. It makes it possible to develop characteristics of the work of people who represent Generation Z.
2. Conceptual categories have been developed that can contribute to creating new paradigms for human resources management that benefit from the competencies of Generation Z.
3. Professionals in human capital management can use the analysis presented on the competency structure of Generation Z staff to improve the quality of public administration management.
4. Interpretations of the mechanism of structural formation of the individual components of the competencies of Generation Z are presented.

## Figures and Tables

**Table 1:** The Values of analysed Gen Z representatives.

Values	Per Cent of All Responses
Family, health, friendship	59.9
Knowledge and skills development	18.4
Professional carrier development and high salary	10.5
Patriotism	0.0
Other	11.2

Source: own elaboration.

**Table 2:** Gen Z representatives and types of social media.

Type of Social Media	Per Cent of All Responses
Facebook	81.4
Instagram	74.8
TikTok	37.2
You Tube	66.7
Other	20.7

Source: own elaboration.

**Table 3:** Social media influence on Gen Z representatives.

Social Media Influence on Gen Z Choices	Per Cent of All Responses
My choices are sometimes guided by what others think about this choice on social media	44.4
I am always guided by what others think about some issue on social media	4.2
My choices are not guided by what others think about this choice on social media	51.4

Source: own elaboration.

**Table 4:** Ethics in workplace.

Answers	Answers (Per Cent of All Responses)
Ethics in the workplace is not the most important.	55.9
Ethics in the workplace is the most important	32.7
Ethics in the workplaces is fiction	5.7
The salary is more important than ethics	1.5

Source: own elaboration.

**Table 5:** Reasons for interest in future work in public administration.

Reasons for Interest in Future Work in Public Administration	Answers (Per Cent of All Responses) *
Due to permanent employment contract	31.5
Due to the legally guaranteed remuneration for work and holidays	53.6
Due to high (according to the surveyed) wages in public administration	7.0
Due to the possibility of implementing the public interest while employed in public administration	20.6
Due to the lack of skills to work in business	7.0

* The respondents could give multiple answers.

**Table 6:** Structure of competencies of Gen Z representatives.

Variable Understudy	Components of Competence (Per Cent) *								
Assessment of competencies *	RT	EA	NS	LS	EB	FL	A	C	CO
	34.1	38.1	38.4	36.0	60.4	60.7	55.9	53.8	76.0

RT—risk-taking, EA—ethical attitudes, NS–knowledge and skills, LS—leadership skills, EB—emotional balance, FL—flexibility, A—activity, C—creativity, CO—commitment. * The respondents could give multiple answers.

## Data Availability

Not applicable.
